# Benefit of Catheter Ablation for Atrial Fibrillation in Heart Failure Patients with Different Etiologies

**DOI:** 10.3390/jcdd10100437

**Published:** 2023-10-20

**Authors:** Songbing Long, Yuanjun Sun, Xianjie Xiao, Zhongzhen Wang, Wei Sun, Lianjun Gao, Yunlong Xia, Xiaomeng Yin

**Affiliations:** 1 Department of Cardiovascular, The Central Hospital of Shaoyang, Shaoyang 422000, China; wylbdhj@163.com; 2 Department of Cardiovascular, The First Affiliated Hospital of Dalian Medical University, Dalian 116011, China; yuanjunsun@126.com (Y.S.); xiaoxianjie0704@sina.com (X.X.); dy_wzz@126.com (Z.W.); se7en43@me.com (W.S.); gaoljmd@126.com (L.G.);

**Keywords:** atrial fibrillation, cardiomyopathy, catheter ablation, pulmonary vein isolation, impaired systolic ejection fraction, heart failure, long-term follow-up

## Abstract

(1) Background: A plethora of studies have elucidated the safety and efficacy of catheter ablation (CA) for patients afflicted with atrial fibrillation (AF) and concomitant reduction in left ventricular ejection fraction (LVEF). Nevertheless, the literature on the benefits of CA in the specific etiological context of heart failure (HF) remains limited. This study delineates a comparative assessment of outcomes for patients with AF and reduced LVEF across the primary etiologies. (2) Methods: Our inquiry encompassed 216 patients diagnosed with congestive heart failure and an LVEF of less than 50 percent who were referred to our institution for circumferential pulmonary vein isolation (CPVI) between the years 2016 and 2020. The selection criteria included a detailed medical history while excluding those suffering from valvular disease, congenital heart disease, and hypertrophic cardiomyopathy. In an effort to scrutinize varying etiologies, patients were stratified into three categories: dilated cardiomyopathy (DCM, n = 56, 30.6%), ischemic cardiomyopathy (ICM, n = 68, 37.2%), and tachycardia-induced cardiomyopathy (TIC, n = 59, 32.2%). (3) Results: Following an average (±SD) duration of 36 ± 3 months, the prevalence of sinus rhythm was 52.1% in the DCM group, 50.0% in the ICM group, and 68.14% in the TIC group (*p* = 0.014). This study revealed a significant disparity between the DCM and TIC groups (*p* = 0.021) and the ICM and TIC groups (*p* = 0.007), yet no significant distinction was discerned between the TIC and ICM groups (*p* = 0.769). Importantly, there were no significant variations in the application of antiarrhythmic drugs or recurrence of procedures among the three groups. The mortality rates were 14.29% for the DCM group and 14.71% for the ICM group, which were higher than the 3.39% observed in the TIC group (DCM vs. TIC *p* = 0.035 (HR = 4.50 (95%CI 1.38–14.67)), ICM vs. TIC *p* = 0.021 (HR = 5.00 (95%CI 1.61–15.50))). A noteworthy enhancement in heart function was evidenced in the TIC group in comparison to the DCM and ICM groups, including a higher LVEF (*p* < 0.001), diminution of LV end-diastolic diameter (*p* < 0.001), and an enhanced New York Heart Association classification (*p* = 0.005). Hospitalization rates for heart failure were discernibly lower in TIC patients (0.98 (0,2) times) relative to those with DCM (1.74 (0,3) times, *p* < 0.01) and TIC (1.78 (0,4) times, *p* < 0.001). Patients with paroxysmal atrial fibrillation and brief episodes were found to achieve superior clinical outcomes through a catheter ablation strategy. (4) Conclusion: Patients diagnosed with TIC demonstrated a more pronounced benefit from catheter ablation compared to those with DCM and ICM. This encompassed an augmented improvement in cardiac function, an enhanced maintenance of sinus rhythm, and a reduced mortality rate.

## 1. Introduction

Atrial fibrillation (AF) is acknowledged to adversely impinge on heart failure (HF) prognosis, effectively doubling mortality rates [1,2,3,4]. The simultaneous manifestation of both conditions in a single patient invariably precipitates a prognosis graver than the occurrence of either malady in isolation [5]. An elevation in resting heart rate and the forfeiture of functional atrial contractility culminate in a truncated diastolic filling period, thereby instigating a diminution in cardiac output. Catheter ablation (CA), a ubiquitous intervention for patients with AF, exhibits success rates approximating 80% [6]. Successful restoration and sustenance of sinus rhythm (SR) through catheter ablation are anticipated to enhance left ventricular ejection fraction (LVEF), left ventricular (LV) dimensions, exercise endurance, and overall quality of life [7,8].

An assembly of controlled trials and retrospective observational analyses has postulated that ablation procedures retain analogous efficacy and safety among patients with heart failure relative to those devoid of such conditions. Within the purview of a randomized multicenter AATAC study undertaken in 2016 [9], the catheter ablation cohort registered a diminished mortality rate (*p* = 0.037) and fewer unforeseen hospital admissions (*p* < 0.001) over a 2-year observation span in contrast to the Amiodarone group. The CASTLE-AF [10] clinical trial and AMICA [11] study illuminated a marked curtailment in mortality and hospitalization for the aggravation of heart failure in patients subjected to ablation. Furthermore, the CABANA subgroup analysis (2021) unveiled significant reductions in mortality, AF recurrences, and an enhancement in quality of life as compared to drug therapy [12]. Bergonti et al. found that patients recovering from LV systolic dysfunction after AF ablation have less wide QRS complexes, less dilated left atria, less frequently a known etiology, and more frequently persistent AF [13]. Nonetheless, the existing literature offers limited insight into the role of atrial fibrillation catheter ablation in the genesis of heart failure. This inquiry explores the long-standing efficacy of cardiac ablation (CA) in patients with heart failure (HF) instigated by three cardinal etiologies: tachycardia-induced cardiomyopathy (TIC), dilated cardiomyopathy (DCM), and ischemic cardiomyopathy (ICM).

## 2. Methods

### 2.1. Patient Population

All procedures conducted in the study involving human subjects adhered to the ethical principles outlined by the institutional and/or national research committee and were consistent with the 1964 Helsinki Declaration, along with its subsequent amendments or equivalent ethical standards. The data collected in this paper have obtained the consent of the parties. Consent was obtained for all data collected in this study. A total of 3012 patients with drug-resistant AF who were treated at the First Affiliated Hospital of Dalian Medical University between May 2016 and April 2020 were included. Among these, 206 patients exhibited symptoms of heart failure and were characterized by a left ventricular ejection fraction (LV) ≤ 50% and New York Heart Association (NYHA) Class ≥ II [14]. Of this subset, 56 were diagnosed with DCM, 68 with ICM, and 59 with TIC; the remaining 25 were excluded from other structural heart disease. The definition of TIC in our study was patients with AF and HF; the reduction of LVEF (HFrEF) is reversible following HF treatment by reverse atrial fibrillation in 6 months [15]. DCM is typified by left ventricular or biventricular dilation and impaired contraction unexplained by abnormal loading conditions or coronary artery disease cardiomyopathy [16]. Ischemic cardiomyopathy (ICM) is delineated as a cardiomyopathy resulting from extended myocardial ischemia, leading to impaired systolic function of the heart.

### 2.2. Baseline Evaluation

All patients underwent transthoracic echocardiography 2 ± 1 days prior to ablation to evaluate left atrium dimensions and LV functions. Transesophageal echocardiography was performed before AF catheter ablation to appraise left ventricular systolic function and eliminate the presence of any intracardiac thrombus. Additionally, 24 h ambulatory electrocardiography was carried out 1–2 days before AF CA to monitor heart rate and rhythm.

### 2.3. Electrophysiological Study and Radiofrequency CA

Oral anticoagulation (OAC) therapy with phenprocoumon was ceased at least one day prior to ablation and replaced by low-molecular-weight heparin. Local anesthesia was administered using lidocaine at all catheter access sites. Following double transseptal puncture access using a modified Brockenbrough technique, patients were heparinized to maintain an activated clotting time > 250 s. Circumferential pulmonary vein (PV) isolation (CPVI) was the initial ablation strategy utilized in all AF patients undergoing ablation; it was complemented by additional substrate modification, encompassing left atrial linear ablation and isthmus ablation. The subsequent catheters were inserted into the femoral vein and strategically positioned: (a) distal 10 poles (Webster Fixed Curve Catheter; Biosense Webster, Diamond Bar, CA, USA) at the coronary sinus; (b) distal 4 poles (Webster Fixed Curve Catheter; Biosense Webster) at the right ventricle; (c) a spiral decapolar mapping catheter (Lasso; Biosense Webster) or a high-density mapping catheter (Pentaray; Biosense Webster) at the targeted PV through SL1 transseptal sheath (Intracardiac Catheter Introducer Kit and Transseptal Needle, Synaptic Medical, Beijing, China); and a 3.5 mm open irrigated tip ablation catheter (ThermoCool SmartTouch; Biosense Webster) in combination with the CARTO mapping system (Biosense Webster) to reconstruct a three-dimensional electroanatomical left atrium through the SL1 transseptal sheath. Selective angiography of each PV using left anterior oblique (LAO) 45 and LAO 40 fluoroscopic views was performed.

### 2.4. CA Protocol

All patients were subjected to CPVI employing irrigated RF at a maximum temperature of 45 °C, a maximal power of 50 W, and an infusion rate ranging from 17 to 25 mL/min, with the power restricted to 40 W at the posterior wall. The CPVI was executed using a Lasso or Pentaray catheter, with the endpoint defined as the absence of any discernible PV spike potential in the mapping catheter inside lateral PVs. Atrial tachycardia (AT) was managed with LA linear lesions. If frequent premature atrial beats or AT were elicited from the LA posterior wall or SVC, procedures such as LA posterior wall isolation or SVC isolation were implemented. Cavotricuspid isthmus line ablation (CTI ablation) was conducted if any common atrial flutter was evident before or during the procedure. Complex fractionated atrial electrograms (CFAE) guided by an LA CFAE map were executed if SR was not attainable post-CPVI. If AF endured, SR was reinstated by cardioversion with two biphasic DC shocks (150 and 200 J). For recurrent AF or AT cases, further ablation procedures were undertaken, along with SVC isolation, CTI ablation, and focal ablation for AT, to accomplish a bidirectional block.

### 2.5. Medical Therapy Strategies

All patients were administered OAC therapy for a period of 2–3 months subsequent to the procedure and extended to 6 months if CHA2DS2-VASc ≥ 2, with subsequent cessation at the discretion of the general practitioner. HF treatment was performed according to each physician’s decision including thiazide diuretic, spironolactone, tolvaptan diuretics, angiotensin-converting enzyme inhibitors (ACEI), angiotensin II receptor antagonists (ARB), beta-blockers, or digoxin.

### 2.6. Post-Ablation Follow-Up

Anticoagulant therapy post-procedure continued for a minimum of 3 months with non-vitamin K antagonist oral anticoagulants (NOACs) or warfarin, if warfarin targeted an international normalized ratio of 2 to 3 [15]. Then, OAC therapy was prescribed based on the patient’s CHA2DS2-VASc score. After ablation, a transthoracic echocardiogram (TTE) was performed to exclude pericardial effusion, and then on the second day, a 24 h Holter recording was used to assess heart rhythm and heart rate. Medicine therapy was recommended for 3 months after the procedure in patients who remained in SR. The recurrence of AF was assessed according to symptoms, with a 12-lead electrocardiogram, 24 h ambulatory monitoring (3, 6, 9, and 12 months), and, thereafter, at 6-month intervals. A TTE was performed in all patients at the same time to evaluate LV function. Recurrence of AF/AT was defined as recurrent symptoms and documented AF/AT lasting > 30 s on electrocardiogram or 24 h ambulatory monitoring after a 3-month blanking period following ablation.

### 2.7. Statistical Analysis

Categorical variables were depicted as counts and percentages, and continuous variables were articulated as a mean ± SD. Comparisons between two groups were achieved with the Student *t*-test and among three groups, with analysis of variance, if suitable, or the Kruskal–Wallis test otherwise. Alterations between two time points (baseline and follow-ups) were analyzed using paired t-tests or the Wilcoxon signed-rank test on continuous variables, while categorical variables were assessed with the chi-square test or Fisher’s exact test. Survival rates were estimated via the Kaplan–Meier method and compared by the log-rank test, or the Wilcoxon test was applied as needed. Univariate and multivariate analyses with logistic regression were utilized to scrutinize variables for predicting procedural success, reported as odds ratio (OR) with 95% confidence intervals (CIs). Significance tests were two-tailed, and a *p*-value of <0.05 was deemed indicative of statistical significance. All statistical analyses were conducted using Stata 13.0 (StataCorp, College Station, TX, USA) and GraphPad Prism version 9.0 (GraphPad Software Inc., La Jolla, CA, USA).

## 3. Results

### 3.1. Basic Characteristics of Patients

Table 1 summarizes the fundamental characteristics of the three evaluated groups. The patients recruited for this study had an average age of 61.8 ± 8.7 years, and no statistical difference was found among the three groups (*p* = 0.06). The distribution of males was statistically uniform across the groups (*p* = 0.61). All categories of AF were equally represented within the three cohorts (*p* = 0.24), comprising 91 (49.73%) persistent AF patients, 40 (21.86%) longstanding-persistent AF patients, and 50 (27.32%) paroxysmal AF patients, with an average AF duration of 75.97 ± 57.8 days (*p* = 0.09). Efforts to achieve SR with AAD (antiarrhythmic drug) (*p* = 0.48) or electrical cardioversion (*p* = 0.48) were analogous among the groups. Concurrent heart diseases such as valvular disease, congenital heart disease, and hypertrophic cardiomyopathy were excluded. The three groups demonstrated a similar NYHA class (*p* = 0.06), LVEF (*p* = 0.89), LV end-diastolic diameter (*p* = 0.29), and left atrial dimension (LAD, *p* = 0.13). A significant difference was observed in the mean of the resting heart rate (RHR) before ablation and admission RHR among the three groups (*p* < 0.01). The mean of the resting heart rate (RHR) before ablation and admission RHR among the three groups was significantly different (*p* < 0.01). Medical therapy for HF did not differ statistically across the groups.

### 3.2. Maintenance of SR in the Different Etiology of HF

**Figure 1:**
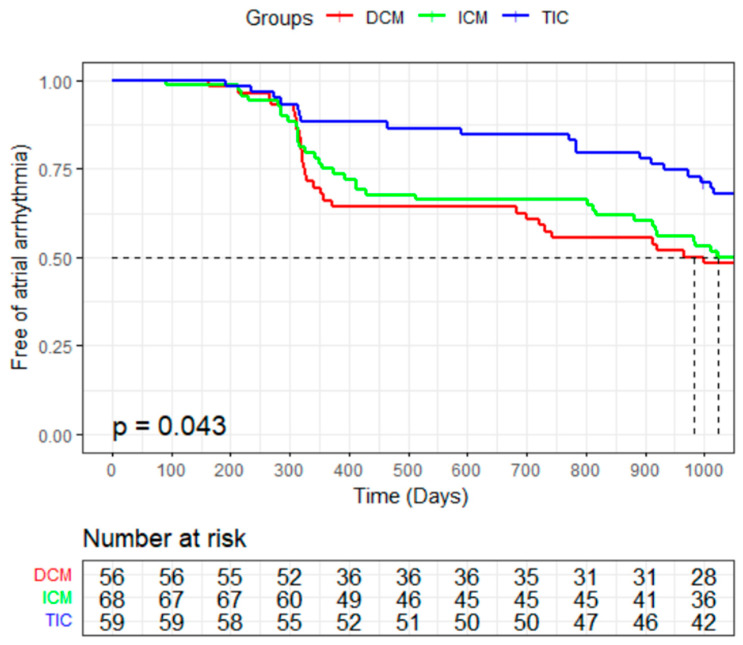
Kaplan–Meier graph of maintenance of the sinus rhythm during the follow-up.

During the follow-up period of 36 ± 2 months (range: 35–37 months), no patients were recorded as lost. Second operations were conducted in 14 patients (25.0%) in the DCM group, 14 patients (20.1%) in the ICM group, and 12 patients (20.3%) in the TIC group (*p* = 0.79). The remaining patients declined a second ablation for diverse reasons. At the end of the follow-up, the TIC group exhibited a higher percentage of patients remaining in SR compared to the DCM group (39/57 (68.4%) vs. 25/48 (52.1%), *p* = 0.021) and ICM group (39/57 (68.4%) vs. 29/58 (50.0%), *p* = 0.007). No statistical difference was discerned between the DCM and ICM groups (25/48 (52.1%) vs. 29/58 (50.0%), *p* = 0.83). Survival analysis via the Kaplan–Meier test estimated SR maintenance across the three groups, as delineated in Figure 1.

### 3.3. HF Hospitalizations, Stroke, and Death

**Figure 2:**
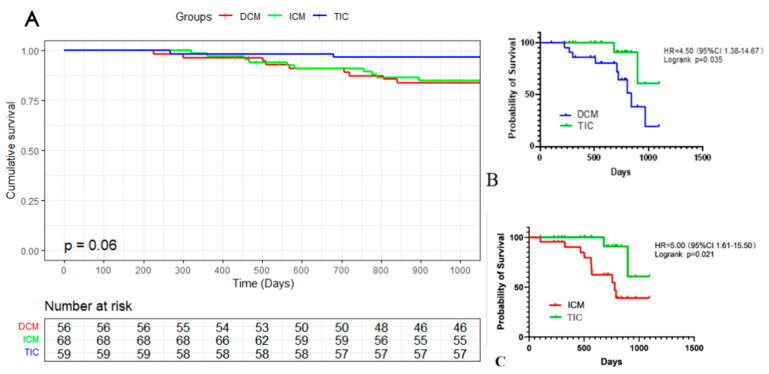
Kaplan–Meier graph of accumulated survival rate. (**A**) Graph of accumulated survival rate among three groups; (**B**) graph of accumulated survival rate between DCM and TIC; (**C**) graph of accumulated survival rate ICM and sTM.

At 36 months post-ablation, all groups demonstrated a significant decrease in HF hospitalization compared to the 30 months prior (*p* < 0.001). HF hospitalization was notably lower in the TIC group (0.98 (0,2) times) than in the DCM (1.74 (0,3) times, *p* < 0.01) and ICM (1.78 (0,4) times, *p* < 0.001) groups, as illustrated in Figure S1A. Stroke occurred in 14/183 (7.6%) patients during the follow-up (FU) period (3 patients in the DCM group, 9 in the ICM group, and 2 in the TIC group), with all patients adhering to oral anticoagulants. At the final follow-up, OAC was discontinued in 57 (35.0%) patients. The mean resting heart rate at the end of the follow-up among the three groups was not significantly different (*p* = 0.13), as shown in Figure S1B. Figure 2 presents the occurrence of deaths in 14.29% of the patients in the DCM group and 14.71% in the ICM groups, which is significantly more than the 3.39% in the TIC group (DCM vs. TIC *p* = 0.035 (HR = 4.50 (95%CI 1.38–14.67)), ICM vs. TIC *p* = 0.021 (HR = 5.00 (95%CI 1.61–15.50))).

### 3.4. Improvements in Heart Function

**Figure 3:**
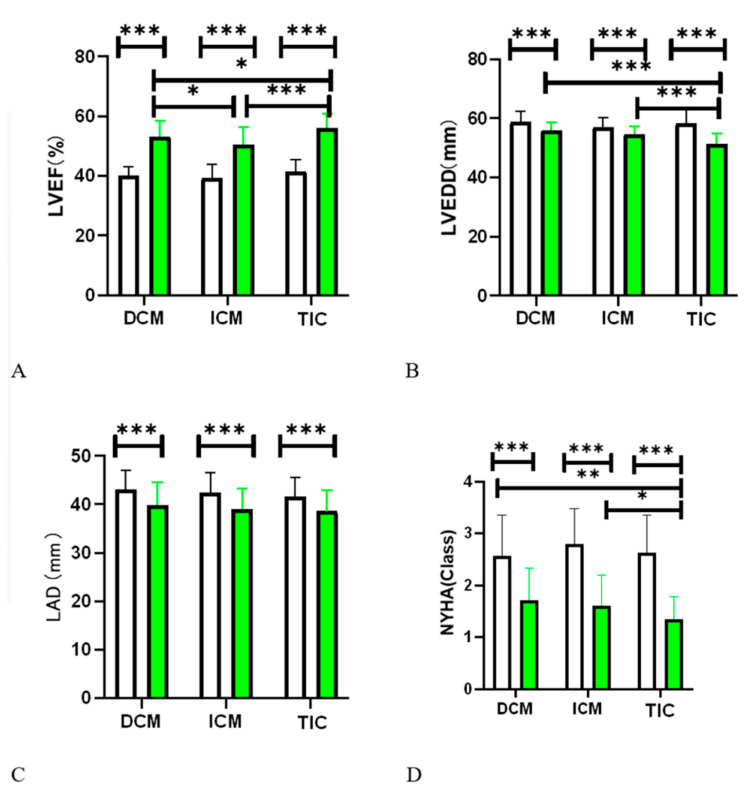
Improvements of heart function between baseline and after up to 30-month follow-up. (**A**) Left ventricular ejection fraction (LVEF); (**B**) left ventricular end-diastolic dimension (LVEDD); (**C**) left atrial dimension (LAD); (**D**) New York Heart Function classification (NYHF). * *p* < 0.05; ** *p* < 0.01; *** *p* < 0.001. The white columns represent the initial baseline data parameters, the green columns represent the finally data parameters.

Following a 36-month period of observation, transformations in heart function are depicted in Figure 3. Utilizing paired tests between baseline and the 36-month follow-up, the Left Ventricular Ejection Fraction (LVEF) and New York Heart Association (NYHA) classification manifested considerable improvements across the three study groups (*p* < 0.001) (Figure 3A). The left ventricular end-diastolic dimension (LVEDD) and left atrial diameter (LAD) underwent statistically significant reductions (*p* < 0.001) (Figure 3B,C).

At the conclusion of the follow-up, patients within the TIC group (56.1 ± 4.8%) exhibited the most pronounced enhancement in LVEF compared to the DCM (53.1 ± 5.4%) (*p* < 0.05) and ICM (50.0 ± 5.8%) (*p* < 0.001) groups, alongside a statistical significance between DCM and ICM groups (*p* < 0.05) (Figure 3A). A significant reduction in LVEDD was observed in the TIC group (51.8 ± 3.6 mm) compared to the DCM (56.0 ± 2.6 mm) (*p* < 0.001) and ICM (54.0 ± 2.7 mm) (*p* < 0.001) groups at the end of the follow-up. Meanwhile, the LAD remained consistent across the three groups at the follow-up’s termination (DCM 39.9 ± 4.7 mm vs. ICM 39.0 ± 4.3 mm vs. TIC 38.6 ± 4.4 mm) at the end of the follow-up (*p* = 0.40). Variations within the three groups at distinct follow-up durations are illustrated in Figure S2.

The LVEF (57.5 ± 5.2%) in the TIC group with SR maintenance improved more than those without SR maintenance (55.2 ± 4.7%) (*p* < 0.01), but the LVEDD reduced similarity whether the procedure was successful or not. The LVEF (55.3 ± 5.6%) was more improved in the DCM group with SR maintenance than those without SR maintenance (52.1 ± 5.2%) (*p* < 0.01); the LVEDD was more reduced in patients with SR maintenance (54.2 ± 3.5 mm) than those without SR maintenance (57.6 ± 4.1 mm) (*p* < 0.01) in the ICM group (52.2 ± 5.1% vs. 48.7 ± 4.6%, *p* < 0.01) (52.1 ± 2.5 mm vs. 55.9 ± 3.5 mm, *p* < 0.01). The LVEF was more improved in the TIC group (57.5 ± 5.2%) with SR maintenance than in the DCM (55.3 ± 5.6%) group and the ICM group (52.2 ± 5.1%) (*p* < 0.01) with SR maintenance, and there was a similarity in the reduction of LVEDD (TIC 51.0 ± 3.8 mm vs. DCM 54.2 ± 3.5 mm vs. ICM 52.1 ± 2.5 m) (*p* < 0.05).

By the follow-up’s conclusion, the NYHA class in the TIC group demonstrated marked amelioration relative to the DCM (*p* < 0.001) and ICM groups (*p* < 0.01) (Figure 3D), yet improvements between the DCM and ICM group were not statistically significant (*p* = 0.38).

### 3.5. Catheter Ablation in HF Patients with Different Types of Atrial Fibrillation

Patients were stratified into three discrete groups, with each corresponding to a different type of atrial fibrillation. The foundational characteristics of these patients are delineated in Table 2 and Table S1. Notably, the average age in the paroxysmal group (56.2 ± 9.5 years) was found to be younger than in both the persistent (62.9 ± 7.5 years) and long-standing persistent groups (66.5 ± 6.3 years) (*p* < 0.01). Concurrently, the duration of atrial fibrillation in the paroxysmal group (43.6 ± 28.5 months) was abbreviated in comparison to the persistent (86.2 ± 49.6 months) and long-standing persistent (117.2 ± 50.3 months) (*p* < 0.01) groups. Following the designated follow-up period, the persistent group evidenced the most marked improvement in LVEF (54.1 ± 5.3%), as opposed to the paroxysmal group (52.4 ± 5.3%) (*p* < 0.05), with no discernible variation between the persistent and long-standing persistent groups (53.2 ± 4.4%) (*p* = 0.4). The LVEDD was significantly attenuated in the paroxysmal group (53.6 ± 3.3) compared to the long-standing persistent group (55.1 ± 3.2 mm) (*p* < 0.05), although no statistical significance emerged when juxtaposed with persistent patients (53.7 ± 3.7 mm) (*p* = 0.85) at the follow-up’s conclusion. The NYHA class (1.35 (2,4)) exhibited more pronounced enhancement in the paroxysmal group relative to the persistent (1.59 (2,4)) and long-standing persistent (1.81 (2,4)) (*p* = 0.002) counterparts.

In the final assessment, the paroxysmal group (41/56 (73.2%)) maintained a greater proportion of patients in SR as compared to the other two cohorts (*p* < 0.001), and the persistence of SR in the persistent group (49/91 (53.8%)) outstripped that of the long-standing persistent groups (11/36 (30.6%), *p* = 0.018). Mortality in the paroxysmal group (2/54(3.6)) was mitigated relative to the persistent group (15/91(16.5)) (*p* = 0.02), whereas the mortality rates in the long-standing groups were not statistically distinguishable from the other two groups at the termination of follow-up. Neither LAD nor stroke rates manifested statistical significance among the three groups at the study’s end.

### 3.6. Multivariable Analysis

Upon multivariable analysis, factors such as the baseline heart rate in AF patients (HR 3.06 (2.73–5.84), *p* < 0.01), the hospital admission rates in the preceding 36-month period owing to cardiac decompensation (HR 1.06 (0.73–0.84), *p* = 0.046), advanced age (HR 1.03 (0.66–0.80), *p* = 0.02), enlarged LA diameter (HR 1.36 (1.21–1.52), *p* = 0.002), longstanding-persistent AF (HR 1.09 (0.87–0.99), *p* = 0.002), and the duration of AF history (HR 1.06 (0.61–0.87), *p* = 0.001) demonstrated association with the success rate of maintaining SR.

Additionally, the total span of AF history (HR 1.26 (0.91–1.87), *p* = 0.049), longstanding-persistent AF patients (HR 1.39 (1.17–2.99), *p* = 0.042), NYHA class at baseline (HR 1.52 (1.16–2.76), *p* = 0.046), and female gender (HR 2.42 (1.36–2.52), *p* = 0.032) were significantly correlated with overall mortality.

## 4. Discussion

### 4.1. Main Findings

In this investigation, the effectiveness of catheter ablation (CA) for atrial fibrillation (AF) in disparate etiologies of heart failure (HF) was retrospectively scrutinized. The findings revealed the following: (1) subsequent to an average follow-up period of 3 years, patients with tachycardia-induced cardiomyopathy (TIC) manifested a significantly enhanced rate of long-term sinus rhythm (SR) maintenance compared to those in dilated cardiomyopathy (DCM) and ischemic cardiomyopathy (ICM) cohorts; (2) cardiac function ameliorated markedly following CA across all groupings, albeit TIC exhibited greater improvements in left ventricular ejection fraction (LVEF), New York Heart Association (NYHA) classification, and diminution of left ventricular end-diastolic dimension (LVEDD); (3) the TIC group experienced reduced mortality and HF-related hospital admissions in comparison with DCM and ICM groupings; and (4) individuals afflicted with paroxysmal AF and HF appear to accrue augmented benefits subsequent to CA.

### 4.2. Improvement of Cardiac Function by CA in AF with TIC

Distinguishing patients diagnosed with TIC from those in the incipient stages of DCM frequently poses challenges as both conditions may culminate in cardiomyopathy. Nonetheless, survival rates amongst TIC patients were notably superior to those observed in cases of DCM or ICM, especially in instances of AF compounded with HF. Consequently, the application of CA in patients characterized by TIC is correlated with a more propitious prognosis and warrants recommendation.

Research pertaining to the clinical aftermath following CA in AF patients with TIC remains scant. Ullah et al. [17] reported suboptimal post-CA SR maintenance in persistent AF patients accompanied by HF in contrast to those devoid of HF over a 3.6-year monitoring period. Within their study, the TIC subgroup failed to exhibit a more favorable outcome relative to other HF etiologies, possibly owing to a diminutive sample size of 18 TIC patients, rendering the statistical potency relatively weak. In contrast, Calvo et al. [18] contrasted CA outcomes in TIC with non-TIC patients, revealing SR retention rates of 40% and 60% subsequent to single and multiple interventions in their TIC cohort throughout a 2-year observation span, respectively. Seigo Y et al. [19] asserted that persistent AF patients with TIC achieve a more favorable prognosis post-CA versus those without CA. Rillig A et al. [20] observed a notably augmented enhancement in left ventricular (LV) systolic function in individuals with TIC alongside long-term SR preservation. Our inquiry further substantiates that TIC confers a superior clinical advantage over DCM and ICM. In our research, neither the resting heart rate (RHR) prior to ablation nor the admission RHR of TIC exhibited extreme tachycardia, which is potentially attributable to the prevalence of ventricular rate control medication amongst the patients.

Within the realm of TIC, SR sustenance markedly exceeds that witnessed in both DCM and ICM, which is a phenomenon partially explicable by the significant elevation in LVEF. While the enhancement of LVEF and contraction of LVEDD were congruent in DCM and ICM subjects, these parameters were conspicuously higher in TIC cases. This discrepancy may elucidate the pronounced reduction in NYHA class within the TIC population relative to the DCM and ICM cohorts. Intriguingly, a pronounced betterment in cardiac function was evident during early follow-up (initial 12 months) in those with TIC, whereas subsequent follow-up (conclusion of 12 months) did not reveal significant further improvements.

### 4.3. Long-Term Outcome after AF Ablation in Patients with Reduced LVEF

Numerous investigations have championed the employment of catheter ablation in patients suffering from HF and AF, with notable findings including a 56% mitigation in hospital admissions and a 49% relative diminution in overall mortality [12,21,22]. Our study corroborates the insights gleaned from prior research. Following CA, cardiac function exhibited amelioration across the three distinct etiological groups. Furthermore, there was a decline in the clinical incidence of hospital admissions attributable to cardiac decompensation subsequent to ablation. In our cohort, oral anticoagulation (OAC) was terminated in 57 patients (35%). A continuance of OAC for a minimum of three months post-ablation was advocated, with further recommendations aligned with the CHA2DS2-Vasc score. A higher incidence of stroke was observed in patients who discontinued OAC relative to those persisting with OAC. The utilization of OAC following radiofrequency (RF) ablation of AF demonstrates considerable variation, but our findings suggest the advisability of maintaining OAC in patients with elevated CHA2DS2-Vasc scores. A meta-analysis showed that catheter ablation reduces mortality and the occurrence of heart failure hospitalizations [23]. Pallisgaard et al. [24] found that patients with HF first seem to have a worse prognosis; the development of HF before AF was associated with a higher rate of death compared to AF before HF and AF and HF occurring within 30 days [24]. Mortality manifested more frequently in DCM and ICM subjects than in TIC individuals in our analysis. The primary rationale may be the superior rate of SR retention in TIC and the more pronounced enhancement in cardiac function in comparison to both groups. Deutekom [25] considered that exploring the temporality of AF and the different LVEF-based subtypes of HF and its prognostic impact would be needed in the future.

### 4.4. Efficacy of CA in SR Maintenance

Despite the incorporation of extensive substrate-based procedures in addition to pulmonary vein isolation (PVI), overall success rates remain moderate; however, numerous studies have reported an arrhythmia-free survival exceeding 70% at the conclusion of the follow-up period [10,23,24,26]. The highest survival rate, as reported by Hunter et al. [27], reached 92%. In the CASTLE-AF study, the SR maintenance rate was 75% across 37 months of follow-up [25,27]. Within our research, the SR maintenance rate was 57%, and 40 patients (21.9%) underwent repeat procedures. The success rate in our study was inferior to previous reports, which is mainly attributable to the elevated prevalence of patients with persistent atrial fibrillation and ischemic cardiomyopathy, as well as an older demographic with prolonged durations of atrial fibrillation.

Patients diagnosed with TIC exhibited the paramount rate of SR maintenance, at 68.4% relative to DCM and ICM, which is a finding that resonates with antecedent research [8,19]. Subjects with paroxysmal AF recorded arrhythmia-free survival rates exceeding 70%, and shorter AF durations correlated with higher SR maintenance rates within our study. The baseline heart rate of AF patients was found to be linked with AF recurrence post-CA. A protracted duration of AF typically results in a decreased ventricular rate and compromised atrial function, which are factors that may contribute to AF recurrence.

### 4.5. Benefits of CA in Different Types of AF

Extensive research has illuminated that paroxysmal AF accrues superior benefits subsequent to CA in comparison to persistent and long-standing persistent AF [10,19,22,26,27]. Within our analysis, the age cohort experiencing paroxysmal AF was characterized by younger demographics, and the duration of AF was comparatively abbreviated relative to the other two classifications. Moreover, the rate of SR maintenance exhibited a notable elevation in cases of paroxysm. The implementation of CA in HF demonstrated a more favorable prognosis and diminished mortality in the context of paroxysmal AF as opposed to non-paroxysmal AF, particularly among subjects with a limited history of AF. Such evidence proposes that the tactical selection of therapeutic interventions for AF within HF patients necessitates individualized consideration.

### 4.6. Limitations

Our investigation is encumbered by its non-randomized, single-center constitution with a retrospective methodology. The cohort was relatively constrained, and the duration of follow-up was not expansive. Efforts to attenuate bias included the alignment of three distinctive etiological groups of HF patients by age, gender, and AF type. The juncture of diagnosis for diminished EF remained inaccessible to patients, thereby potentially influencing the quantification of augmented cardiac function and success rates. While ambulatory monitoring was employed to gauge AF recurrence, it may inadvertently have led to an inflated estimation of success. Reiterative assessments of endpoints also contributed to the study’s limitations. Although our definition of tachycardia-induced cardiomyopathy (TIC) was predicated on contemporary consensus guidelines, TIC’s diagnosis was made retrospectively, and dilated cardiomyopathy (DCM) was determined through exclusion based on medical history. The inability to perform cardiac MRI and left ventricular global longitudinal strain (GLS) in our cohort might engender certain inaccuracies in outcomes. This single-center investigation, with its non-randomized and retrospective character, might not furnish evidence as robust as multi-centered, randomized controlled trials, yet it offers significant insights into real-world clinical applications.

## 5. Conclusions

Patients afflicted with tachycardia-induced cardiomyopathy demonstrated elevated rates of SR maintenance and decreased hospital admissions, a reduction in mortality, and enhanced improvements in cardiac function relative to those with dilated cardiomyopathy or ischemic cardiomyopathy. Furthermore, short-duration paroxysmal AF yielded superior clinical outcomes when managed with a CA strategy.

## Figures and Tables

**Table 1:** Baseline characteristics of patients.

Variable	DCM	ICM	TIC	*p*
**Clinical characteristics** Age—year	(n = 56) 61.8 ± 7.6	(n = 68) 63.9 ± 8.3	(n = 59) 59.5 ± 9.6	0.06
Male, n (%)	33 (58.9)	34 (50.0)	32 (54.2)	0.61
Classification of atrial fibrillation—no. (%)				
Paroxysmal	19 (33.9)	14 (20.6)	23 (38.9)	0.07
Persistent	23 (41.1)	40 (58.8)	28 (47.5)	0.13
longstanding-persistent	14 (25.0)	14 (20.6)	8 (13.6)	0.30
Duration of atrial fibrillation—month	71.2 ± 44.7	85.6 ± 52.4	69.4 ± 37.2	0.09
Previous electrical cardioversion—no. (%)	17 (30.4)	26 (38.2)	17 (28.9)	0.48
No. of antiarrhythmic drugs tried	46 (82.1)	56 (82.4)	44 (74.6)	0.48
Treatment with amiodarone—no. (%)	6 (10.7)	2 (2.9)	5 (8.5)	0.22
Hypertension—no. (%)	21 (37.5)	35 (51.5)	21 (35.6)	0.14
Diabetes mellitus, n (%)	8 (14.3)	18 (26.5)	9 (15.3)	0.15
CHA 2 DS 2-Vasc-score (median)	2.38 (1,4)	3.91 (2,6)	2.05 (1,4)	<0.01
hospital admission rates (during the 30-month before) (median)	3.09 (0,6)	3.07 (0,5)	2.46 (0,4)	0.05
Resting heart rate (admission)	107.2 ± 20.1	101.6 ± 23.6	138.7 ± 24.1	<0.01
Resting heart rate (bpm) (before ablation)	95.6 ± 19.6	86.3 ± 22.7	102.6 ± 21.7	<0.01
**Heart function**				
Baseline LVEF (%)	40.0 ± 3.0	39.2 ± 4.6	41.1 ± 4.0	0.89
LVEDD at baseline (mm)	58.8 ± 3.7	57.0 ± 3.2	58.3 ± 4.7	0.29
LAD at baseline (mm)	43.1 ± 4.0	42.4 ± 4.1	41.6 ± 4.0	0.13
NYHA functional class at baseline (median)	2.57 (2,4)	2.79 (2,4)	2.63 (2,3)	0.06
**medical therapy for HF**				
thiazide diuretic, n (%)	56 (100)	68 (100)	59 (100)	1.00
spironolactone, n (%)	56 (100)	68 (100)	59 (100)	1.00
tolvaptan diuretics, n (%)	8 (14.3)	9 (13.2)	6 (10.2)	0.78
ACEI/ARB, n (%)	51 (91.1)	57 (83.8)	49 (83.1)	0.39
Beta-blockers	50 (89.3)	58 (85.3)	56 (94.9)	0.21
Digoxin	10 (17.9)	16 (23.5)	8 (13.6)	0.35

Abbreviations: AF, atrial fibrillation; CA, catheter ablation; CHA2DS2-VASc, congestive heart failure, hypertension, age ≥ 75 (doubled), diabetes, stroke (doubled), vascular disease, age 65–74, and sex category (female); DCM, dilated cardiomyopathy; HF, heart failure; LV, left ventricular; NYHA, New York Heart Association. ACEI, angiotensin-converting enzyme inhibitors; ARB, angiotensin II receptor antagonists.

**Table 2:** Baseline characteristics in different types of AF.

Variable	Paroxysm	Persistent	Longstanding-Persistent	*p*
**Clinical characteristics** Age—yr	(n = 56) 56.2 ± 9.5	(n = 91) 62.9 ± 7.5 *	(n = 36) 66.5 ± 6.3 **	<0.01
Male, n (%)	32 (57.1)	51 (56.0)	18 (50.0)	0.77
Cardiomyopathy—no. (%)				
DCM	19 (33.9)	23 (25.3)	14 (38.9)	0.26
ICM	14 (25.0)	40 (44.0)	14 (38.9)	0.07
TIC	23 (41.1)	28 (30.8)	8 (22.2)	0.15
Duration of atrial fibrillation—mo	43.6 ± 28.5 *	86.2 ± 49.6 ^#^	117.2 ± 50.3 **	<0.01
Hypertension—no. (%)	22 (39.3)	38 (41.8)	17 (47.2)	0.75
Diabetes mellitus, n (%)	7 (12.5)	20 (22.0)	8 (22.2)	0.32
CHA 2 DS 2 -Vasc-score (median)	2.34 (1,6)	2.96 (1,6) *	3.39 (1,6) **	<0.01
Hospital admission rates (during the 36-month before) (median)	3.09 (0,5)	2.84 (0,6)	2.75 (0,4)	0.48
**Heart function**				
Baseline LVEF (%)	40.5 ± 5.0	40.2 ± 3.5	39.4 ± 3.5	0.45
LVEDD at baseline (mm)	57.5 ± 4.2	57.9 ± 4.0	58.9 ± 3.5	0.24
LAD at baseline (mm)	42.7 ± 5.2	42.3 ± 3.6	41.8 ± 3.0	0.54
NYHA functional class at baseline (median)	2.73 (2,4)	2.65 (2,4)	2.67 (2,4)	0.63
**At the end of FU**				
LVEF at last FU (%)	52.4 ± 5.3	54.1 ± 5.2 *	53.2 ± 4.4	0.14
LVEDD at last FU (mm)	53.6 ± 3.3	53.7 ± 3.7	55.1 ± 3.2 **	0.10
LAD at last FU (mm)	39.1 ± 5.6	38.9 ± 4.1	40.0 ± 2.9	0.48
NYHA functional class at last FU	1.35 (2,4)	1.59 (2,4) *	1.81 (2,4) **	0.002
hospital admission rates during the FU (median)	1.4 (0,3) **	1.46 (0,4) ^#^	2.05 (0,2)	<0.01
**Procedures and complications**				
All pulmonary veins isolated—no. (%)	56 (100)	91 (100)	36 (100)	1.00
Additional left atrial linear ablation—no. (%)	2 (3.6)	46 (50.5) *	22 (61.1) **	<0.01
Total duration of radiofrequency ablation—min	83.5 ± 24.4	88.7 ± 22.6	96.0 ± 33.6	0.29
Total duration of fluoroscopy—min	5.3 ± 3.2	5.9 ± 2.3	6.4 ± 2.3	0.18
Total duration of procedure—min	164.6 ± 51.6	170.5 ± 39.70	190.9 ± 43.2	0.13
Serious complications—no. (%)				
Tamponade	1 (1.8%)	4 (4.4%)	3 (8.3%)	0.06
stroke	0 (0%)	0 (0%)	0 (0%)	1.00
Repeat operations—no. (%)	11 (19.6)	22 (24.2)	7 (19.4)	0.18
Overall stroke—no. (%)	2 (3.6%)	9 (9.9%)	3 (8.3%)	0.36
Overall success—no. (%)	41 (73.2) *	49 (53.8) **^#^**	11 (30.6) **	<0.01
Overall death—no. (%)	2 (3.6) *	15 (16.5)	4 (11.1)	0.06

Abbreviations: AF, atrial fibrillation; CA, catheter ablation; CHA2DS2-VASc, congestive heart failure, hypertension, age ≥ 75 (doubled), diabetes, stroke (doubled), vascular disease, age 65–74, and sex category (female); DCM, dilated cardiomyopathy; HF, heart failure; LV, left ventricular; NYHA, New York Heart Association. FU, follow-up. * *p* < 0.01 paroxysm vs. persistent; ** *p* < 0.01 paroxysm vs. longstanding-persistent; ^#^ *p* < 0.01 persistent vs. longstanding-persistent.

## Data Availability

The data that support the findings of this study are available from the corresponding author upon reasonable request.

## Supplementary Materials

The following supporting information can be downloaded at: https://www.mdpi.com/article/10.3390/jcdd10100437/s1. Table S1: Procedures and complications; Figure S1. HF hospitalization between baseline and after up-to 30-month follow-up. * *p* < 0.05; ** *p* < 0.01, *** *p* < 0.001.
