# Supplementary material for: Benefit of Catheter Ablation for Atrial Fibrillation in Heart Failure Patients with Different Etiologies
Source: J Cardiovasc Dev Dis. 2023 Oct 20;10(10):437. doi: 10.3390/jcdd10100437 (PMC10607920; doi:10.3390/jcdd10100437)
Supplement: Supplementary file 1 [file jcdd-10-00437-s001.zip › jcdd-2596446-supplementary.pdf]

Table S1. Procedures and complications

Variable	DCM	ICM	TIC	P
All pulmonary veins isolated — no. (%)	56（100）	68（100）	59（100）	1.00
Additional left atrial linear ablation — no. (%)	20（35.7）	30（44.1）	20（34.5）	0.45
Total duration of radiofrequency ablation — min	86.7±24.4	89.1±22.6	93.0±33.6	0.49
Total duration of fluoroscopy — min	6.5±3.2	5.2±2.3	5.2±2.3	0.09
Total duration of procedure — min	174.6±51.6	173.5±39.70	176.9±43.2	0.94
Serious complications — no. (%)				
Tamponade	3（5.4%）	4（5.9%）	1（1.9%）	0.47
stroke	0（0%）	0（0%）	0（0%）	1.00
Repeat operations — no. (%)	14（25.0）	14 (20.1）	12（20.3）	0.79
Overall success — no. (%)	25（52.1）	28（50.0）	39（66.1）	＜0.01

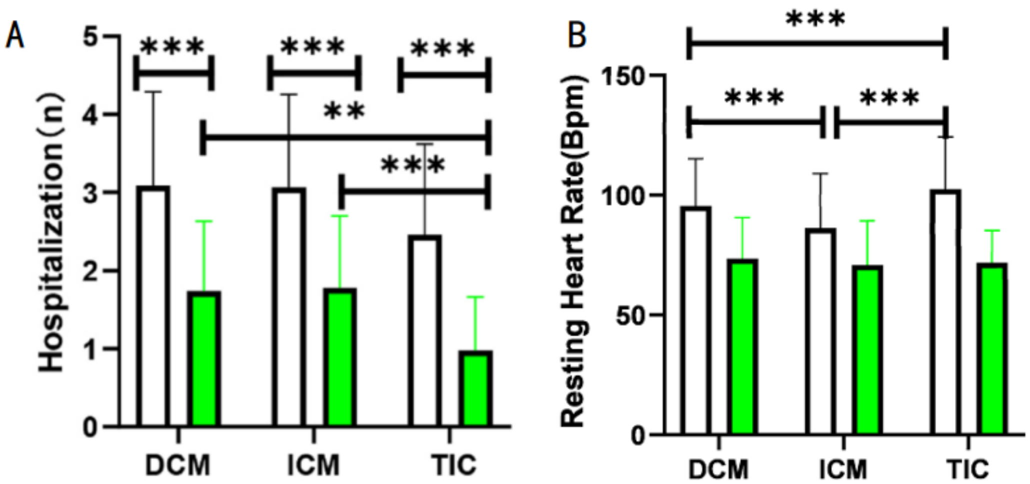

Figure S1. A. HF hospitalization between baseline and after up-to 30-month follow-up; B. Resting heart rate between baseline and after up-to 30-month follow-up. * $P < .05$; ** $P < .01$; *** $P < .001$;

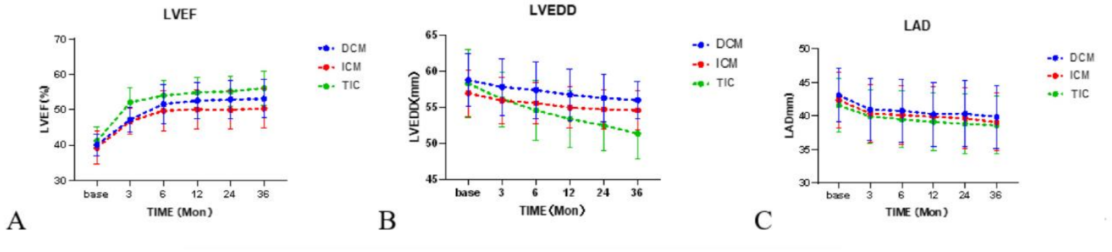

Figure S2. The changes of the three groups at different follow-up times. A. Left ventricular ejection fraction (LVEF); B. Left ventricular end of diastolic dimension (LVEDD); C. Left atrial dimension (LAD);
